# Editorial: Pediatric Long-Term Non-invasive Ventilation

**DOI:** 10.3389/fped.2021.654578

**Published:** 2021-02-22

**Authors:** Brigitte Fauroux, Renato Cutrera

**Affiliations:** ^1^Pediatric Non-invasive Ventilation and Sleep Unit, Paris University EA 7330 VIFASOM (Vigilance Fatigue Sommeil et Santé Publique), Necker University Hospital, Paris, France; ^2^Respiratory Unit and Pediatric Sleep & Long Term Ventilation Unit, Pediatric Hospital Bambino Gesù, IRCCS, Rome, Italy

**Keywords:** OSAS (obstructive sleep apnea syndrome), obesity, cystic fibrosis—CF, CPAP (continuous positive air pressure), BiPAP (bilevel positive airway pressure), neuromuscolar disorders, palliative care—methods, long term non-invasive ventilation

Long-term non-invasive mechanical ventilation consists in the delivery of non-invasive mechanical ventilation (NIV) at least 6 h per day for more than 3 weeks (1). Long term NIV is increasing world-wide, with broader indications, and is associated with an increase in survival rate in children with chronic conditions. In this Research Topic different experts in the field deepen the topic of long-term NIV in children.

Praud reviewed the current use of home NIV. He focused on the advantages of long-term home NIV compared to prolonged hospitalization and to home invasive ventilation in different countries. He reported interesting data of two scoping reviews about this topic (2, 3). In addition, Basa et al. have brought to the topic their experience in a middle-income country. Their experience could be considered as a model of setting up a home mechanical ventilation center: since 2001 the number of patients on ventilation has increased rapidly. They reported their numbers and their high proportion of invasively ventilated patients (about a half of the patients). As can be seen from other works in literature, the increase in NIV experience is associated with a reduction of invasively ventilated patients (4).

Certainly, NIV is increasing its popularity in the last few years due to its relative simplicity. However, technical knowledge of machines, interfaces, circuits as well as indications is required. Ferreira deepened the topic of interfaces and circuits. NIV is usually well-tolerated but may be associated with some side effects. An experienced team confident with tricks and possible complications related to NIV is crucial to avoid NIV failure. Main adverse events are discomfort, skin lesions, unintentional leaks, or disturbed facial growth (midface hypoplasia). On the one hand, skin lesions and unintentional leaks may be reduced by changing interface periodically, using protective dressings and selecting the appropriate mask type. On the other hand, discomfort may be reduced using active humidification finally improving adherence (5, 6). Pavone et al., provided a comprehensive report of different ventilators available on the market and the different ventilatory modes. Nowadays, new hybrid modes are flanking the traditional pressure-target and volume-target modes. Hybrid modes require more studies in pediatrics but the small literature available shows that they may reduce asynchronies and facilitate management of children poorly adapted to traditional modes (7). Moreover, new ventilators are equipped with built-in software and allow remote monitoring of ventilation parameters: these data supply information about trends of patients on home ventilation and are useful for the clinician to understand possible causes of suboptimal ventilation (8). It is evident that new technologies are constantly improving information given to clinicians about home NIV. Those new possibilities are changing type of follow-up of children at home giving. Nowadays, ventilators provide many information, often accessible remotely, and the clinician can monitor ventilator trends easily and without requiring hospitalization. Khirani et al. provided a comprehensive analysis of available literature about follow-up of patients on NIV. Unfortunately, there is a lack of validated indications about strategies to monitor patients on CPAP or NIV. However, the authors reported their extensive experience with patients on home CPAP or NIV: type of follow-up is variable depending on the team experience, underlying disease and patient's condition. Certainly, CPAP and NIV require a regular assessment with a minimum time (1–3 months after first adaptation and reassessment every 6–12 months) (9). Monitoring should include ideally a P(S)G [poly(somno)graphy] or nocturnal pulse oximetry (SpO_2_) and capnography (CO_2_) recording, if P(S)G is not available. As stated above, monitoring of home patients has been improved during the last few years with new tools such as telemedicine and analysis of data obtained from built-in software. New home ventilators provide useful information on built-in software (adherence, leaks, efficacy of ventilation) representing a useful tool for the clinician for monitoring ventilation. Caution must be taken about interpretation of data as no specific pediatric software is available and there is a lack of standardization of interpretation of data.

As reported by Praud, long-term NIV is increasingly used worldwide, including developing countries, and provides improvements in terms of decreased mortality and increased quality of life. The increased use of NIV in different settings has expanded indications of its use. The use of NIV has been extended in the last few years to patients with bronchopulmonary dysplasia (BPD) requiring long-term respiratory support. Interestingly, despite the paucity of studies to support the use of NIV in patients with cystic fibrosis (CF) and non-CF bronchiectasis, the application of NIV seems to stabilize the decrease of lung function when the disease is advanced (10). Finally, other studies support the use of NIV in patients with bronchiolitis obliterans, making extremely wide the range of possible indications of NIV. Nevertheless, it seems crucial to highlight that in such circumstances an experienced team is required to select carefully patients that may benefit from NIV. Nevertheless, it is crucial to monitor patients in order to evaluate quickly possible NIV failure to change approach accordingly with the main disease. Paglietti et al. reviewed the indication of NIV in children with central hypoventilation (CCHS). In the past, invasive ventilation was the only way to avoid hypoventilation and the subsequent increase of serum carbon dioxide related to the impairment of respiratory drive. The increase of experience in the management of NIV accompanied by the better knowledge of these disorders has meant that an increasing number of children with CCHS has been switched to NIV when feasible (patients requiring ventilation only during sleep). Moreover, decannulation of children with CCHS along with the transition from invasive ventilation to NIV is nowadays to be considered if the patient require only nocturnal ventilatory assistance (11).

The topic goes through the other indications of NIV. Fauroux et al. reported in their paper the most recent evidences about the use of NIV in children with neuromuscular disorders (NMD). Pump failure due to neuromuscular impairment is often an indication of ventilatory support and experience about this topic is increasing worldwide. Therefore, children affected by NMD may experience hypoventilation and this condition could determine mild cognitive impairment, daytime fatigue, poor sleep quality and difficult to carry out normal activities. No validated criteria to start long-term home NIV are available for clinicians, but is currently widely agreed that NIV should be initiated when nocturnal hypoventilation is present, even before the occurrence of symptoms. Therefore, is very important that centers who take care of NMD children are equipped with adequate equipment (polygraphy, SpO_2_ and transcutaneous CO_2_ monitoring) and experienced professionals to early diagnose nocturnal hypoventilation in order to start and monitor NIV appropriately. However, Fauroux et al. recognized that benefits of NIV in children with NMD are not well-defined: a limited number of studies are available in literature with very small sample size. Nevertheless, NIV showed to be associated with prolonged survival and an improvement in the quality of life in children with Duchenne muscular dystrophy (12) and prolonged survival in children with spinal muscular atrophy type 1 (13). The heterogeneity of disorders, ages and outcomes determines the importance of the correct follow-up for each patient for optimal medical care.

Another of the most represented indication is the use of NIV in children with severe obstructive sleep apneas (OSA). Verhulst reviewed the use of NIV and continuous positive airway pressure (CPAP) in children with obesity and Down syndrome. These children have a high incidence of residual OSAS after adenotonsillectomy (AT) and consequently require additional treatment. CPAP and NIV represent a second level of treatment, when not only adenotonsillectomy has failed, but also weight loss, anti-inflammatory medications and eventual orthodontics treatments are not effective. Interestingly, both those categories of children are affected by low adherence to ventilation and high drop-out rates of therapy. Patients with Down syndrome showed improvement in adherence with time; an alternative in patients with complete intolerance could be high flow nasal cannula as showed by Amaddeo et al. (14).

NIV or CPAP failure requires a multidisciplinary approach considering the underlying diagnosis, comorbidities and eventual alternative treatments (surgery, weight loss, etc.). Amaddeo et al. reviewed possible alternatives to NIV approach in different conditions (NMDs, OSAS, central nervous system diseases etc.). When no treatments are feasible or effective and the ventilatory balance is seriously impaired, invasive ventilation via tracheotomy should be considered. As far as ventilation via tracheostomy is associated with several complications and require a comprehensive multidisciplinary discussion, it represents in many cases a safe management of patients with chronic respiratory failure when no other choices are available. Interestingly, is open the debate about the right choice of ventilation in children dependent to ventilator 24 h per day. If on the one hand, some groups consider NIV feasible, on the other invasive ventilation is considered safe and may improve quality of life when time spent on NIV is too high (15, 16).

Nevertheless, in children with severe underlying diseases, the choice between invasive and non-invasive approach rises to an important ethical dilemma and clinicians must consider all the different aspects in order to choose the better mode of ventilation. Krivec and Caggiano analyzed the use of NIV for palliative purposes. Pediatric palliative care is an emerging field characterized by the difficult balance between prolonged suffering and relief symptoms. Therefore, on the one hand medical and technological advances have made long-term support and survival possible in children with severe chronic health conditions, on the other this may result in prolonging suffering. Authors highlighted the paucity of studies about this actual and crucial topic. They proposed an interesting ethical framework for decision making in pediatric long-term non-invasive ventilation. This approach may provide appropriate room for the interaction between the involved patients, their families, healthcare providers, health authorities and the broader society in order to use NIV to relief symptoms and not to prolong suffering (17).

Another unexpected result of improving medical care is the need to ensure a smooth process of transition to adult care for children with chronic and complex diseases who reach adulthood. Notably, children on long-term NIV are often patients with complex needs with a limited or absent independence. Onofri et al. dealt with this issue providing different care models of transition to adult care used for other chronic pediatric diseases (congenital heart disease, cystic fibrosis, type 1 diabetes mellitus). They discussed barriers and facilitators to the process of health care transition to adulthood. Ideally, the process should be uninterrupted, well-coordinated and comprehensive. However, on the one hand young adults on NIV are vulnerable, prefer pediatric supportive setting and may be scared about the passage to adult healthcare system. On the other hand, adult services may be not familiar with chronic pediatric conditions and adult physicians could lack of experience with patients with poor autonomy. This underestimated and multifaceted issue requires information, education and communication for adolescents, their families and healthcare providers. However, currently no transition model has been shown to be superior to another (18).

## Conclusions

This series offers a comprehensive discussion about main indications, complications, newest evidences and main clinical issues for children on long-term NIV. These reviews written by experts in the field represent a useful tool for clinicians in everyday clinical practice. Moreover, this collection is a worth set of different point of views about different crucial issues for children on NIV and ideas for further researches.

## Author Contributions

RC and BF contributed equally to the conception and drafting of the editorial. All authors contributed to the article and approved the submitted version.

## Conflict of Interest

The authors declare that the research was conducted in the absence of any commercial or financial relationships that could be construed as a potential conflict of interest.
